# Supplemental dietary Selenohomolanthionine affects growth and rumen bacterial population of Shaanbei white cashmere wether goats

**DOI:** 10.3389/fmicb.2022.942848

**Published:** 2022-10-20

**Authors:** Long-Ping Li, Lei Qu, Tuo Li

**Affiliations:** ^1^Shaanxi Provincial Engineering and Technology Research Center of Cashmere Goats, Yulin University, Yulin, China; ^2^College of Life Sciences, Yulin University, Yulin, China

**Keywords:** Shaanbei white cashmere goat, Selenohomolanthionine, rumen bacterial communities, nutrient digestibility, growth, high-throughput sequencing

## Abstract

Selenium (Se) is an important trace element for all livestock growth. However, little is known about the dietary supplementation of Selenohomolanthionine (SeHLan) effect on growth and rumen microbiota of cashmere goats. In this study, thirty-two growing Shaanbei white cashmere wether goats with mean body weight (26.18 ± 2.71) kg were randomly assigned into 4 treatments, each with 8 replicates. The goats in 4 experimental groups were fed the basal diet (0.016 mg/kg Se) added with organic Se in the form of SeHLan, namely, control group (CG, added 0 mg/kg Se), low Se group (LSE, added 0.3 mg/kg Se), medium Se group (MSE, added 0.6 mg/kg Se), and high Se group (HSE, added 1.2 mg/kg Se). The feed experiment lasted for 70 days including 10-day adaptation, followed by 11 days digestibility trial including 7-day adaptation and 4-day collection period. On the last day of feeding experiment, rumen fluid was collected for microbial community analysis. The feed, orts, and fecal samples were collected for chemical analysis during digestibility trial. The results showed that average daily feed intake (ADFI) and the apparent digestibility of crude protein (CP) were both quadratic ally increased with increased SeHLan supply (*P_*quadratic*_* < 0.05), while average daily gain (ADG) and feed conversion ratio (FCR) showed a linear response (*P_*linear*_* < 0.05). The ADFI and ADG were all highest in the MSE group, which also had the lowest FCR (*P* < 0.05). Alpha diversity indices of the microbial community did not differ among four treatments. While principal coordinates analysis (PCoA) showed that rumen bacterial population differed among four groups. Taxonomic analysis revealed that Bacteroidetes, Firmicutes, and Euryarchaeota were the dominant phyla. The dominant families were Prevotellaceae, Selenomonadaceae, Methanobacteriaceae, and Bifidobacteriaceae. The significantly different rumen bacterial genera were found to be *Methanobrevibacter*, *Quinella*, *Christensenellaceae_R-7_group*, *Veillonellaceae_UCG-001*, and *Succinivibrionaceae_UCG-002* (*P* < 0.05). In addition, Tax4fun analysis revealed that SeHLan supplemented groups enhanced the enrichment of genes related to energy metabolism, amino acid metabolism, carbohydrate metabolism, and enzymes. Twenty-eight pathways showed significant differences among four treatment groups (*P* < 0.05). In conclusion, dietary supplementation of medium SeHLan significantly affects rumen bacterial composition and ultimately promotes Shaanbei white cashmere wether goats nutrient digestibility and growth.

## Introduction

Ruminants have a taxonomically diverse microbiota in their rumen, which is the most efficient natural fermentation system. Rumen microorganism are mainly composed of bacteria, fungi, archaea, protozoa, and a small amount of viruses, which are essential for the conversion of plant cell wall and fibrous substances into absorbable compounds such as proteins and volatile fatty acids (VFAs) through the fermentation through rumen microorganisms (Dai et al., 2015). Diets and additives directly affect the number and viability of rumen microorganisms (Zheng et al., 2021). A lot of studies have confirmed that trace elements affect gut health by regulating gut microbiome community structure (Faulkner et al., 2017; Biscarini et al., 2018; Ishaq et al., 2019). Selenium (Se), essential microelement as a component of glutathione peroxidase (GSH-Px), can protect cell membranes from peroxide damage (Cobanova et al., 2017). In addition, Se is also critical for the biosynthesis of coenzyme A/Q, conversion of Thyroxine (T4) to Triiodothyronine (T3), tricarboxylic acid cycle (TCA), and electron transport in the respiratory chain, which will promote improvement in the nutrient digestibility and animal growth performance (Hefnawy et al., 2010).

Supplementation of Se in the diet can effectively improve the antioxidant status of rumen microorganisms, thereby promoting rumen microbial growth and rumen fermentation, improve the apparent digestibility of nutrients, and ultimately promote the animal growth and production (Shi et al., 2011; Wang et al., 2019). Cui et al. (2021) reported that dietary addition of Se-enriched yeast (SeY) can effectively increase the NH_3_-N, total VFA (TVFA), acetate, butyrate, and propionate concentrations in Tibetan sheep. In addition, dietary SeY supplementation improved the Corriedale lambs ruminal straight-chain and iso-branched-chain VFA (Miltko et al., 2016). Moreover, Wei et al. (2019) found that dietary supplementation of Se promoted rumen fermentation and the apparent digestibility of crude protein (CP), neutral detergent fiber (NDF), and acid detergent fiber (ADF) in dairy cows. It is also reported that adding 0.3 mg/kg of SeY to the diet increased the milk yield of dairy cows (Wang et al., 2009). Alimohamady et al. (2013), Wang et al. (2019) also demonstrated that dietary Se supplementation increased the digestibility of dry matter (DM), OM, CP, NDF, and ADF in lambs. Furthermore, using high-throughput sequencing technology, Cui et al. (2021) found that SeY-supplemented diet significantly affected the relative abundance of *Prevotella 1*, *Rikenellaceae RC9 gut group*, *Ruminococcus 2*, *Lachnospiraceae XPB1014 group*, *Carnobacterium*, *Hafnia-Obesumbacterium*, and KEGG pathways of carbohydrate and amino acids in Tibetan sheep. Zhang et al. (2020) designed primers and performed real-time PCR assay to study the ruminal microbiota in lactation dairy cows; the results showed that sodium selenate-supplemented diet not only increased the abundance of amylolytic bacteria included *Ruminococcus* and *Fibrobacter*, but also promoted the activity of cellobiase, carboxymethyl cellulase, xylanase, and protease. Mihaliková et al. (2005) observed that supplementation of Se (sodium selenite or SeY) in diet significantly affected the ruminal ciliate populations of *Ophryoscolex* and *Diploplastron* in sheep. Therefore, it was hypothesized that the diet of Shaanbei white cashmere goats supplementation with Se may promote carbohydrate metabolism by altering the rumen microflora, subsequently improving the growth performance of Shaanbei white cashmere goats. However, the effects of supplemented Se on the ruminal symbionts in goats remain poorly characterized. The microbial mechanism underlying the improvement in growth performance of Shaanbei white Cashmere goats supplemented with Se needs to be further studied. To the best of our knowledge, limited study has focused on identifying and understanding the diversity of ruminal microorganism of dietary Se supplementation using high-throughput sequencing technology, and this requires further in-depth research and investigation.

The Shaanbei White Cashmere goat, an important local breed in Northern China, produces both excellent cashmere and high-quality meat, which is bred by the cross of Liaoning Cashmere goat (♀) and Shaanbei Ziwuling Black goat (*male*). It is well adapted to the harsh natural environments and strong resistance to roughage, cold, sand, and some disease (Liu, 2018). The numbers of Shaanbei White Cashmere goat in Northern China are 10 million and composition of the main source of income for local farmers. In China, 71.6% of the counties and districts are Se-deficient areas and Shaanxi province belongs to the area with Se severe deficiency (the content of forage Se less than 0.05 mg/kg). Studies have shown that the content of Se in the soil of the Shaanbei white cashmere goat feeding area and the Se content in the blood of Shaanbei white cashmere goat are 0.039 mg/kg and 0.020 mg/L, respectively, indicating that the Se contents from soil to forage grass are lower than the recommended level (0.05 mg/kg) (Xiong, 2012). Selenium deficiency often causes white muscle disease, muscle atrophy, and reduce animal production levels (Mehdi and Dufrasne, 2016). Therefore, it is necessary to supplement Se through feed additives for Shaanbei White Cashmere goats. Dietary Se supplementation mainly includes organic forms of Se (SeY, selenomethionine, selenocysteine, Nano-Se, HMSeBA, namely, 2-hydroxy-4-methylselenobutanoic acid, etc.) and inorganic forms of Se (selenite, selenate, selenide, etc.). Compared with inorganic Se, organic Se has low toxicity, high safety, and efficiency, and has been widely used in animal production. Selenohomolanthionine [4,4′-selenobis (2-aminobutanoic acid), SeHLan] is a kind of organic Se which is biosynthesized by Candida utilis with simple metabolic pathway and more efficient synthesis of selenoproteins than selenomethionine (Tsuji et al., 2010). However, as a new type of organic Se, studies on SeHLan in livestock are rarely reported.

Previous studies on the effects of dietary Se supplementation for ruminants mostly focused on its deficiency diseases, immune function, and production performance. However, few studies reported the effects of dietary Se supplementation on the population and structure of ruminant microorganisms. In this study, we evaluated the effects of dietary SeHLan supplementation on growth and bacterial community structure of Shaanbei white cashmere wether goats. The results of this study will broaden our understanding of the effect of Se on rumen bacterial diversity and richness, and provide a scientific basis for determining the appropriate dosage of SeHLan supplementation to improve the health and productivity of Shaanbei white cashmere goats.

## Materials and methods

### Ethics statement

All animal procedures were approved by the Animal Care and Use Committee of Yulin University (Yulin, China) and were in accordance with the university’s guidelines for animal research (file no.: YLU2021-2).

### Animal, diets, and experimental design

Thirty-two Shaanbei white cashmere wether goats with similar body [(26.18 ± 2.71) kg] were selected and randomly assigned into 4 treatment groups with 8 replicates in each group. The basic diet containing 0.016 mg/kg DM Se as control diet (control group, CG) and SeHLan was added to three treatments based on the control diet to make the contents of Se as follows: low Se group (LSE: 0.3 mg/kg DM), medium Se group (MSE: 0.6 mg/kg DM), and high Se group (HSE: 1.2 mg/kg DM). Taking into account the Se contents of the basal diet, the actual Se contents of the four diets were 0.016, 0.316, 0.616, and 1.216 mg/kg DM, respectively. SeHLan was obtained from AB Agri Pumeixin Tech (Jiangxi) Co., Ltd. (Jiangxi, China). Se concentration in basal diet was determined with some modifications (Alonso et al., 2005). In brief, after the samples were digested using microwave digester with 10 mL of concentrated nitric acid and 2 mL of hydrogen peroxide (30%), the mixture was cooled at room temperature and transferred to a volumetric flask (50 mL) until analysis. The Se concentration in digested samples was determined using hydride generation atomic fluorescence spectrometer (AFS9300, FuDa Technology Co., Ltd., Shanghai, China). The basal diet was formulated according to Chinese Feeding Standard of Meat-Producing Sheep and Goats (NY/T 816-2004) with the exception of Se (Table 1).

**TABLE 1:** The composition and ingredients of the basal diet.

Items	Content
**Ingredient, (% of DM)**	
Alfalfa hay	10
Corn straw	20
Oat grass	10
Peanut vine	10
Soy straw	10
Caragana	10
Corn	6
Soybean meal	6
Cottonseed mea	11
Wheat bran	5.3
NaCl	0.5
CaHPO4	0.2
Premix_1_	1
Total	100
**Nutrient composition** ^2^	
DM, %	92.36
DE/(MJ/kg)	9.6
CP, %	11.8
EE, %	1.74
NDF, %	49.90
ADF, %	37.20
Ca, %	1.15
TP, %	0.63
Se/(mg/kg)	0.016

^1^The premix provided the following of the diet (per kilogram): VA 1800 IU, VD_3_ 260 IU, VE 23 mg, Zn 30 mg, Mn 25 mg, Fe 50 mg, Cu 11 mg, Co 0.2 mg, I 1 mg.

^2^DE was calculated according to feed ingredient composition on the dry matter basis, while the others were measured values.

DM, dry matter; DE, digestive energy; CP, crude protein; EE, ether extract; NDF, neutral detergent fiber; ADF, acid detergent fiber; Ca, calcium; TP, total phosphorus; Se, selenium.

The feeding experiment period lasted for 70 days including 10 days adaptation and 60 days of sampling and data collection period. Four goats were fed in a separate pen, with free access to water, all animals were fed at 09:00 a.m. and 17:00 p.m., and 5% orts were ensured throughout the experiment. During the feeding experiment, the feed offered and refused of goats in each pen were recorded every day to calculate the average daily feed intake (ADFI), and the ADFI was determined as the feed intake/60. The body weight of all goats were measured at beginning and thereafter at every 15 days internals until 60 days of the trial before the morning feeding and the average daily gain (ADG) was calculated. The feed conversion ratio (FCR) was determined as ADG/ADFI during the experimental period. When 70-day feeding experiment was finished, all experimental goats were subjected to an 11-day digestion and metabolism test, and the pretrial and trial lasted for 7 and 4 days, respectively. Each goat was kept in individual homemade metabolic cage where feces were collected using a collector device equipped with a plastic net including an angled ramp. All goats had free access to water throughout the experiment, and diets supplemented with different SeHLan concentrations were provided manually at 09:00 a.m. and 17:00 p.m.

### Sample collection

On the last day of feeding trial, 20 mL of rumen fluid were collected from each goats after 2 h morning feeding using an oral stomach tube as described previously (Shen et al., 2012). The first 50 mL of rumen fluids in each sample were discarded to remove the potential saliva contamination. Each rumen content sample was immediately sealed in a centrifuge tube and frozen in liquid nitrogen and then stored at −80°C for bacterial population diversity analysis. During the final 7 days of digestibility trial, 5% of feed, orts, and 10% fecal samples of each goat were collected and measured daily. The feed, orts, and fecal samples were dried at 65°C and ground using small pulverizer to pass through a 1-mm screen, stored at −20°C for further analysis of dry matter (DM), CP, neutral detergent fiber (NDF), and acid detergent fiber (ADF).

### Chemical analysis and calculations

The DM content of feed, orts, and feces was determined by drying samples in an oven at 105°C for 24 h, and ash content was determined by incinerating samples at 550°C for 3 h in a muffle furnace. Organic matter (OM) was measured as the difference between DM and ash content. The nitrogen content of the experimental diets, diet refusals, and feces was analyzed with a Hanon K9860 fully Automatic Kjeldahl analyzer produced by Hanon Advanced Technology Group Co., Ltd. (Jinan, China); 6.25 was used as the conversion factor to obtain CP values. The contents of NDF (with heat stable α-amylase) and ADF were determined according to methods described by Van Soest et al. (1991) and the Association of Official Analytical Chemists (method number 973.18 C; Association of Official Analytical Chemists, 1990), respectively. The apparent digestibility of nutrients were calculated using the following equation: Digestibility (%) = [Nutrient intake (g/d)−Fecal output (g/d)]/Nutrient intake (g/d) × 100%.

### DNA extraction, PCR amplification, and sequencing

The CTAB/SDS method was used to extract the total genome DNA of all rumen samples. DNA concentration and purity were monitored on 1% agarose gels. According to the concentration, DNA was diluted to 1 ng/μL with sterile water. The V3−V4 hypervariable region of the 16S rRNA gene was amplified with the following primers 341F (5′-CCTAYGGGRBGCASCAG-3′) and 806R (5′-GGACTACHVGGGTWTCTAAT-3′), which have previously been reported to target both bacteria and archaea but with a higher average matching efficiency for bacteria (Berg et al., 2012). All PCR mixtures contained 15 μL of Phusion^®^ High-Fidelity PCR Master Mix (New England Biolabs), 0.2 μM of each primer and 10 ng target DNA, and cycling conditions consisted of a first denaturation step at 98°C for 1 min, followed by 30 cycles at 98°C (10 s), 50°C (30 s), and 72°C (30 s) and a final 5 min extension at 72°C. Mix an equal volume of 1X loading buffer (contained SYB green) with PCR products and perform electrophoresis on 2% agarose gel for DNA detection. The PCR products were mixed in equal proportions, and then Qiagen Gel Extraction Kit (Qiagen, Germany) was used to purify the mixed PCR products according to the manufacturer’s instructions. Following manufacturer’s recommendations, sequencing libraries were generated with NEBNext^®^ Ultra™ II DNA Library Prep Kit (Cat No. E7645). The library quality was evaluated on the Qubit@ 2.0 Fluorometer (Thermo Fisher Scientific) and Agilent Bioanalyzer 2100 system. Finally, the library was sequenced on an Illumina NovaSeq platform and 250 bp paired-end reads were generated.

### Sequence analysis

The raw 16S rRNA gene sequencing reads were demultiplexed, quality-filtered by fastp version 0.20.0 (Chen et al., 2018), and merged by FLASH version 1.2.7 (Magoè and Salzberg, 2011) with the following criteria: (i) the 300 bp reads were truncated at any site receiving an average quality score of <20 over a 50-bp sliding window, and the truncated reads shorter than 50 bp were discarded, reads containing ambiguous characters were also discarded; (ii) only overlapping sequences longer than 10 bp were assembled according to their overlapped sequence. The maximum mismatch ratio of overlap region is 0.2. Reads that could not be assembled were discarded; (iii) Samples were distinguished according to the barcode and primers, and the sequence direction was adjusted, exact barcode matching, 2 nucleotide mismatch in primer matching. The high-quality clean tag were obtained according to the QIIME 1.9.1 quality controlled process (Bokulich et al., 2013). Sequence analyses was clustered by UPARSE software (V7.0.1001) into operational taxonomic units (OTUs) based on 97% similarity (Edgar, 2013). A representative sequence for each OTU was selected for further annotating the taxonomic information using the Silva Database (Quast et al., 2013) based on the Mothur algorithm (Schloss et al., 2009). Multiple sequence alignment was conducted and normalized using MUSCLE 3.8.31 (Edgar, 2004).

In order to analyze the diversity, richness, and uniformity of the communities in our samples, alpha diversity indices, including Observed_otus, Chao1, Shannon, Simpson, ACE, Good’s coverage and Pielou_e, were all calculated with QIIME (Version 1.9.1) and displayed in R software (Version 2.15.3); principal coordinate analysis (PCoA) was performed to obtain principal coordinates and visualize differences of samples in complex multi-dimensional data based on Bray-Curtis dissimilarity matrices using ade4 package and ggplot2 package in R software (Version 2.15.3). Linear discriminant analysis effect size (LEfSe) with the cutoff of LDA score > 3.0 was performed to find out the biomarkers among four groups using R software (version 2.15.3) with the ggplot package. Furthermore, to study the functions of the communities in the samples and find out the different functions of the communities in the different groups, the Tax4Fun, a software package that predicts the functional capabilities of microbial communities based on 16S rRNA datasets, was used for function annotation analysis. The predicted pathways were identified with the Kyoto Encyclopedia of Genes and Genomes (KEGG) database (Aßhauer et al., 2015). A heatmap of the top 35 most abundant genera predicted KEGG pathways that were constructed using R software (Version 2.15.3) with heatmap package.

### Statistical analysis

Statistical analyses were performed using IBM SPSS Statistics 20. The general linear model (GLM) was used to conduct a one-way analysis of variance for the experimental data of the feed intake, weight gain, digestibility, and the α-diversity indices. The model of statistics is as follows: Y_i_ = μ + α_i_ + ε_i_, in which Y_i_, μ, α_i_, and ε_i_ represented the dependent variable, overall mean, diet effect, and error term, respectively. In this model, diet was considered as a fixed effect, while the animal was considered as a random effect. Linear and quadratic effects were tested using orthogonal polynomial contrasts. The *P*-values of linear and quadratic effects were reported as *P*_*linear*_ and *P*_*quadratic*_, respectively. The Kruskal–Wallis rank sum test was used to compare the bacteria relative abundances among the four treatments using IBM SPSS Statistics 20. Significant difference value was set at *P* < 0.05.

## Results

### Growth performance and nutrient digestibility

Growth performance and nutrient digestibility are shown in Table 2. ADFI and the apparent digestibility of CP increased in a quadratic manner in response to dietary SeHLan supply (*P_*quadratic*_* < 0.05). The apparent digestibility of DM, OM, NDF, and ADF was all both linearly and quadratically increased with increasing dietary SeHLan supplementation (*P_*linear*_* < 0.05, *P_*quadratic*_* < 0.05), while ADG and FCR showed a linear response (*P_*linear*_* < 0.05). The ADFI, ADG, and the apparent digestibility of CP were all highest in the MSE group, which also had the lowest FCR (*P* < 0.05).

**TABLE 2:** Effects of selenium supplementation on growth performance and nutrient digestibility of Shaanbei white cashmere wether goats.

Items	Treatments^1^				SEM	*P*-value^2^	
			
	CG	LSE	MSE	HSE		L	Q
**Growth performance**							
ADFI (g/d)	936.45^b^	920.75^b^	1062.43^a^	992.26^a^	22.95	0.920	0.040
ADG(g/d)	48.33^b^	78.33ab	93.75^a^	75.73ab	7.54	0.014	0.087
FCR	19.38^a^	11.75^b^	11.33^b^	13.10ab	1.69	0.028	0.327
**Nutrient digestibility (%)**							
DM	66.39^b^	70.29^a^	71.00^a^	72.15^a^	0.51	0.028	0.022
OM	47.40^c^	52.47^b^	54.42ab	56.32^a^	0.72	0.021	0.013
CP	65.41^b^	68.36^a^	72.13^a^	71.62^a^	0.67	0.013	0.006
NDF	49.02^c^	51.04^b^	54.73ab	58.31^a^	0.84	0.013	0.006
ADF	42.94^c^	49.20^b^	52.97^a^	56.62^a^	0.94	0.003	0.008

^1^CG, LSE, MSE, and HSE: treatment groups supplemented with Se at 0 (control), 0.30 (low), 0.60 (medium), and 1.2 (high) mg/kg dry matter, respectively.

^2^L, linear effect; Q, quadratic effect.

^a,b,c^In the upper-right corner differ (P < 0.05) if without a common letter.

ADFI, Average daily feed intake; ADG, Average daily gain; FCR, feed conversion ratio; DM, dry matter; OM, organic matter; CP, crude protein; DNF, neutral detergent fiber, ADF, acid detergent fiber.

### Diversity of rumen microbiota

**FIGURE 1:**
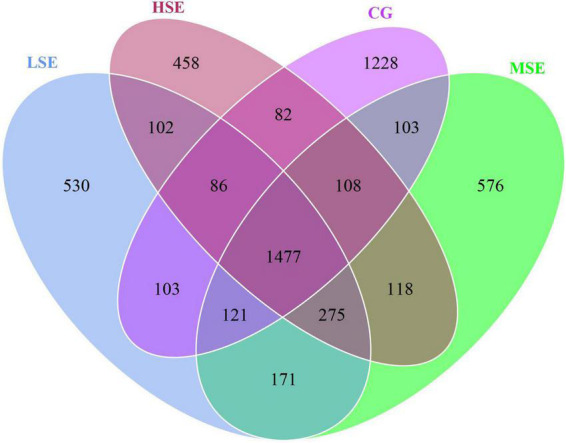
The Venn diagram of the shared and unique OTUs throughout the four different SeHLan added groups. CG, control group; LSE, low Se group; MSE, medium Se group; HSE, high Se group.

**FIGURE 2:**
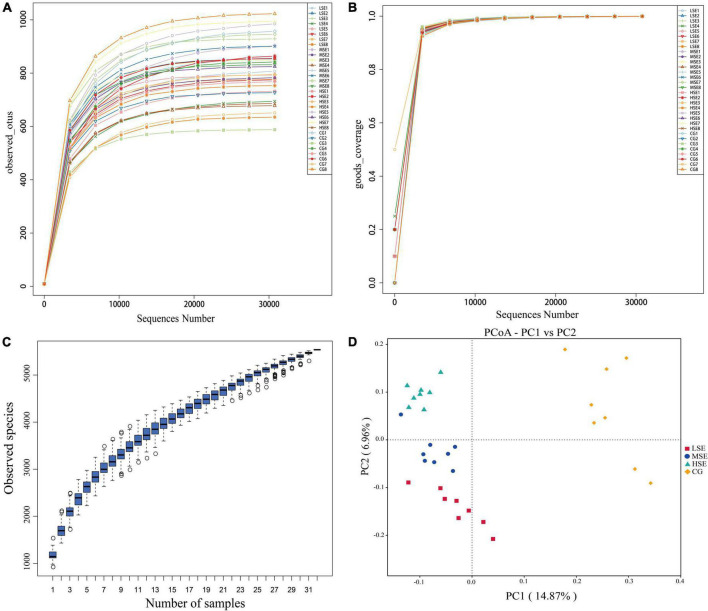
The alpha- and beta-diversity among four SeHLan supplemented groups. **(A)** Rarefaction curves based on V3–V4 of 16S rRNA gene; **(B)** Good’s coverage index; **(C)** Species accumulation curves; **(D)** Principal coordinate analysis (PCoA) plot based on Bray-Curtis distance. CG, control group; LSE, low Se group; MSE, medium Se group; HSE, high Se group.

The 16s rRNA gene sequencing experiment of 32 Shaanbei white cashmere wether goats rumen fluid samples produced a total of 1,687,835 high-quality sequences with an average of 52,745 ± 2556 [(mean ± standard error of the mean (SEM), *n* = 32] per sample. A total of 5,639 OTUs were obtained based on the 97% similarity threshold in the present study. The number of unique OTU in CG, LSE, MSE and HSE groups were 1228 (21.78%), 530 (9.39%), 576 (10.21%), and 458 (8.12%), respectively (Figure 1). The CG group had the highest number of OTUs, and the HSE group had the lowest number of OTUs. 1477 OTUs (52.89% of the total) were shared among four different level SeHLan added groups. The rarefaction curves (Figure 2A) and the Good’s coverage index (Figure 2B) indicated that our sequencing depth is sufficient to contain most of the microbial information and sampling quality met the requirements for sequencing and analysis. Additionally, the species accumulation curves showed that our samples were sufficient for OTUs test and could predict the species richness of samples (Figure 2C). Various alpha-diversity estimators were compared among four treatment groups and it was found that there was no significant difference were observed (*P* > 0.05, Table 3). The PcoA based on Bray-Curtis dissimilarity matrices suggested an obvious separation of the bacterial communities between CG and other three samples (Figure 2D).

**TABLE 3:** Operational taxonomic unit count and α diversity estimation based on the 16S rRNA gene sequencing analysis of the four different treatment groups.

Item	Treatments^1^				SEM	*P*-value^2^	
			
	CG	LSE	MSE	HSE		L	Q
Observed species	801.38	797.75	843.75	814.50	19.21	0.919	0.464
Shannon	7.29	7.63	7.73	7.36	0.11	0.147	0.694
Simpson	0.967	0.976	0.983	0.974	0.004	0.323	0.393
Chao1	805.16	800.99	849.27	821.09	19.56	0.932	0.435
ACE	1385.01	1413.09	1478.34	1477.04	27.93	0.351	0.540
Good’s coverage	0.999	0.999	0.999	0.999	0.0001	0.070	0.715
Pielou_e	0.756	0.792	0.796	0.762	0.009	0.099	0.787

^1^CG, LSE, MSE, and HSE: treatment groups supplemented with Se at 0 (control), 0.30 (low), 0.60 (medium), and 1.2 (high) mg/kg dry matter, respectively.

^2^L, linear effect; Q, quadratic effect.

### Taxonomic composition of rumen microbiota

At the taxonomic level, a total of 2 kingdom (Bacteria and Archaea), 42 phylum, 106 class, 216 order, 288 family, and 415 genus were obtained in all samples from our data. The taxonomical composition of the rumen microbiota was investigated by sequencing the V3−V4 region of the 16S rRNA gene on the rumen samples with the results uploaded to the NCBI’s sequence read archive (PRJNA835512).

**FIGURE 3:**
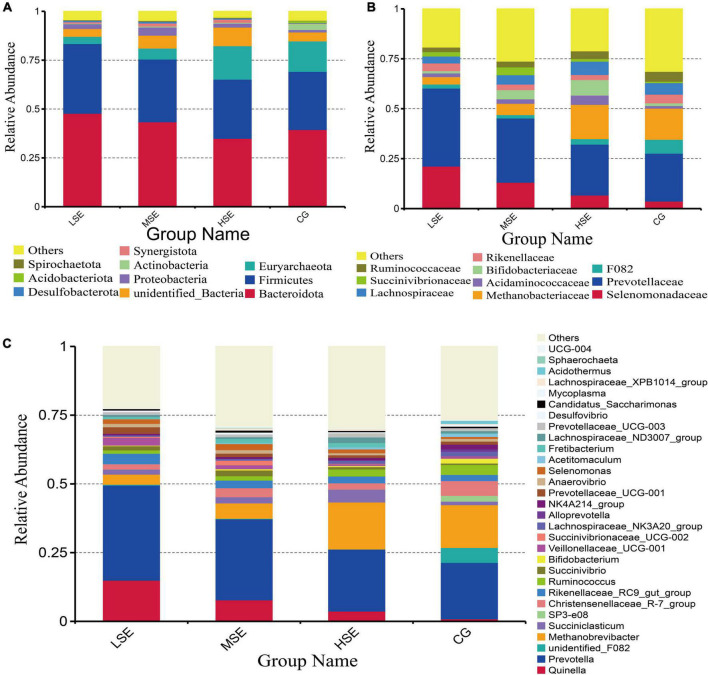
Rumen bacterial compositional profiles of different SeHLan supplemented groups. Relative abundance of bacterial taxa averaged under phylum level **(A)**, family level **(B)**, and genera level **(C)**. The color-coded bar plot shows the average bacterial phylum, family, and genus distributions level of the different diets sampled. CG, control group; LSE, low Se group; MSE, medium Se group; HSE, high Se group.

The dominant bacterial phyla in all four treatment groups include Bacteroidetes and Firmicutes, followed by Euryarchaeota, unidentified_Bacteria, Proteobacteria, Actinobacteriota, and Synergistetes (Figure 3A and Supplementary Table 1). At the family level, Prevotellaceae and Selenomonadaceae were the most highly represented taxa, followed by Methanobacteriaceae, Bifidobacteriaceae, F082, Acidaminococcaceae, Rikenellaceae, Lachnospiraceae, Succinivibrionaceae, Ruminococcaceae, Bacteroidales_RF16_group, and Christensenellaceae (Figure 3B and Supplementary Table 2). At the genus level, among the detected 415 genera, 14 the most abundant genera (average relative abundance of >1% for at least one group) were found in all groups. Three dominant genera (average relative abundance of >5% for at least one group) were *Prevotella*, *Methanobrevibacter*, and *Quinella* in all 4 treatment groups (Figure 3C and Supplementary Table 3).

### Taxonomic differences of rumen microbiota

**FIGURE 4:**
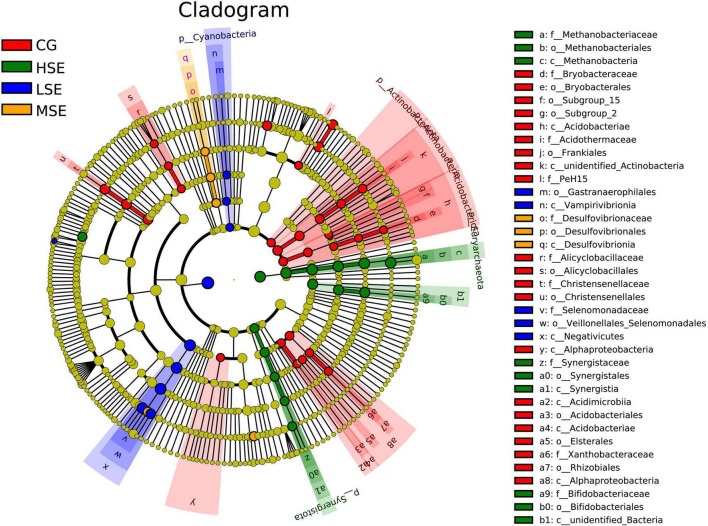
Cladogram of significantly different taxa identified in the rumen microbiome data sets of different SeHLan supplemented groups based on the cut-off of LDA > 3.0 and *P* < 0.05. Clades significantly enriched in each cohort are highlighted by the colors shown in the legend. There are six layers from the inside of this plot to the outside, corresponding to six levels of taxonomy (phylum, class, order, family, genus, and species). The nodes (small circle) with different colors represent bacterial biomarkers of in the corresponding groups with the higher abundance compared with that in the other three groups, while yellow nodes indicate the bacteria that are not statistically and biologically differentially abundant among the four groups. CG, control group; LSE, low Se group; MSE, medium Se group; HSE, high Se group.

**FIGURE 5:**
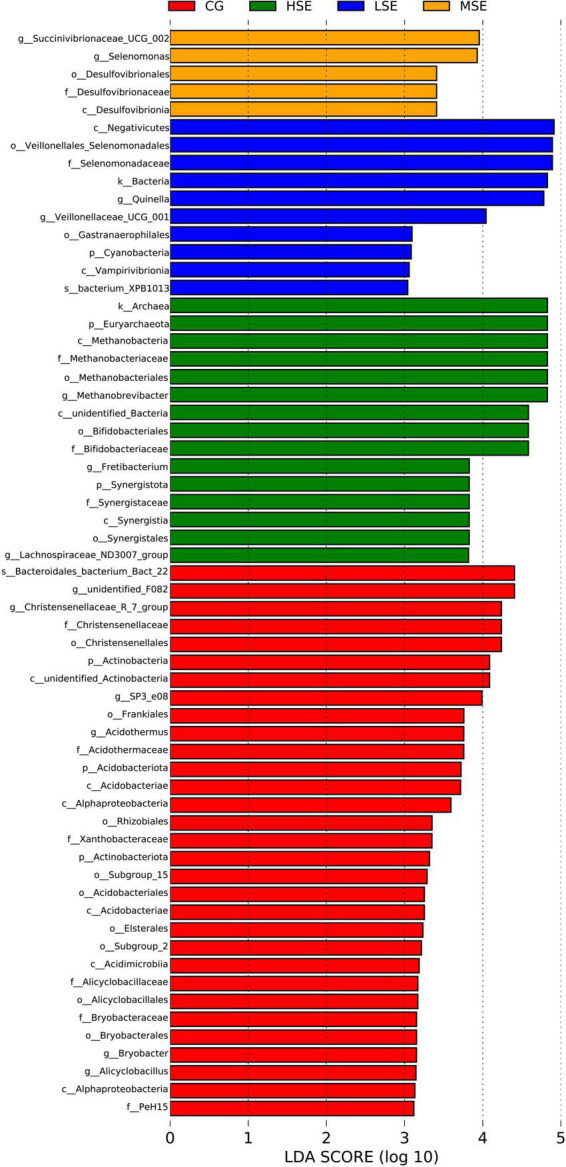
Histogram of the LDA scores computed for differentially abundant rumen bacteria across four treatment groups. Linear discriminant analysis effect size scores could be interpreted as the degree of difference in relative abundance of the analyzed rumen bacteria communities across four groups. The histogram thus identifies which clades among all those detected as statistically and biologically differentially abundant and could explain the features most likely to explain differences across the groups.

For variations of the bacterial taxa composition analysis, bacteria with LDA scores greater than 3.0 and *P* < 0.05 were regarded to have a different abundance and as the biomarkers among four groups with different levels of SeHLan supplementation. Our data showed that twenty-one clades were more abundant in the CG group, five clades were more abundant in the LSE group, three clades were more abundant in the MSE group, and nine clades were more abundant in the HSE group (Figure 4). The Figure 5 depicts the bacterial abundance differences of different level SeHLan supplemented groups. Among them, the most differential bacterial phyla in the rumen of the high-level Se added group (HSE) were Euryarchaeota and Synergistota, while the relative abundance of Actinobacteria, Acidobacteriota, and Actinobacteriota was significantly higher in the rumen of CG group than other three groups which all received SeHLan supplement (LDA > 3.0, *P* < 0.05). At the family level, 3 taxa, including Methanobacteriaceae, Bifidobacteriaceae, and Synergistaceae, exhibited significantly higher abundance in the rumen of HSE group than other 3 groups (LDA > 3.0, *P* < 0.05). Desulfovibrionaceae and Selenomonadaceae are the most differential bacterial families in the MSE and LSE groups, respectively (LDA > 3.0, *P* < 0.05). Six taxa, including Christensenellaceae, Acidothermaceae, Xanthobacteraceae, Alicyclobacillaceae, Bryobacteraceaeand, and PeH15, are the most differential bacterial families in the CG group (LDA > 3.0, *P* < 0.05). At the genus level, we identified 3 taxa as the most differential bacterial genera in HSE group, which could distinguish HSE group from the other 3 groups and were more abundant in the rumen of HSE group samples, including *Methanobrevibacter*, *Fretibacterium*, and *Lachnospiraceae_ND3007_group* (LDA > 3.0, *P* < 0.05). We identified 2 taxa as the most differential bacterial genera in MSE group, which could distinguish MSE group from the other 3 groups and were more abundant in the rumen of MSE group samples, including *Succinivibrionaceae_UCG_002* and *Selenomonas* (LDA > 3.0, *P* < 0.05). Two taxa are the most differential bacterial genera in LSE group, including *Quinella* and *Veillonellaceae_UCG_001* (LDA > 3.0, *P* < 0.05). Six genera, including *unidentified_F082*, *Christensenellaceae_R_7_group*, *SP3_e08*, *Acidothermus*, *Bryobacter*, and *Alicyclobacillus*, are the most differential bacterial genera in CG group (LDA > 3.0, *P* < 0.05). The characteristic bacterial genera with the largest abundance in HSE, MSE, LSE, and CON groups were *Methanobrevibacter*, *Succinivibrionaceae_UCG_002*, *Quinella*, and *unidentified_F082*, respectively.

**FIGURE 6:**
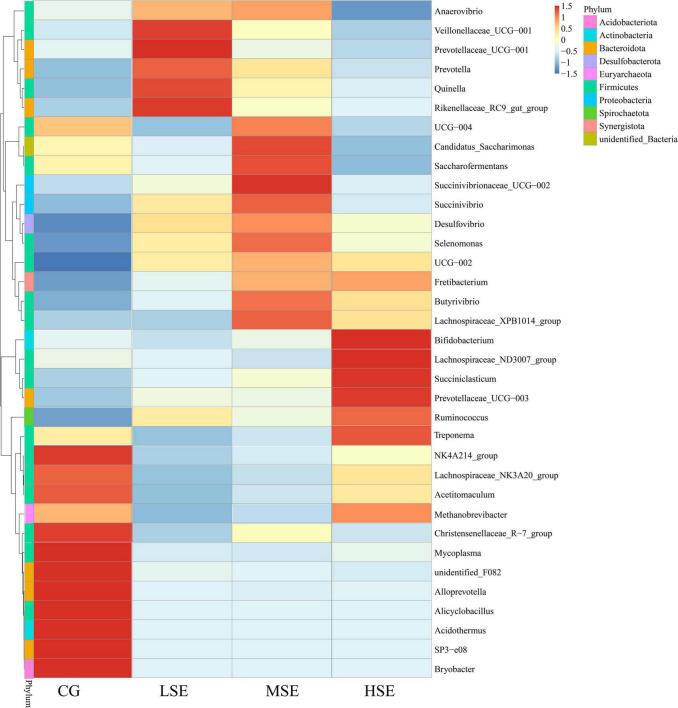
Heatmap analysis of top 35 bacterial genera as determined by the relative abundance of taxonomic genera levels. The vertical axis is sample information, the horizontal axis is species annotation information. On the left is the species clustering tree. The heatmap in the middle corresponds to the standardized Z-score of the relative abundance of each row species.

According to a heatmap (Figure 6), construction based on the relative abundances of top 35 genera can be clearly found that the relative abundances of *NK4A214_group*, *Lachnospiraceae_NK3A20_group*, *Acetitomaculum*, *Christensenellaceae_R-7_group*, *Mycoplasma*, *unidentified_F082*, *Alloprevotella*, *Alicyclobacillus*, *Acidothermus*, *SP3-e08*, and *Bryobacter* were all higher in the CG group than other three groups which were all SeHLan treatment. However, the relative abundances of *Veillonellaceae_UCG-001*, *Prevotellaceae_UCG-001*, *Quinella*, and *Rikenellaceae_RC9_gut_group* were correlated positively with the LSE group (low level Se); the abundances of *Candidatus_Saccharimonas*, *Saccharofermentans*, *Succinivibrionaceae_UCG-002*, *Succinivibrio*, *Selenomonas*, *Butyrivibrio*, and *Lachnospiraceae_XPB1014_group* were correlated positively with the MSE group (medium level Se) and the HSE group (high level Se) correlated positively with the relative abundances of *Bifidobacterium*, *Lachnospiraceae_ND3007_group*, *Treponema*, *Succiniclasticum*, *Prevotellaceae_UCG-003*, *Ruminococcus*, and *Methanobrevibacter*.

### Prediction of rumen microbiota functional pathways

**FIGURE 7:**
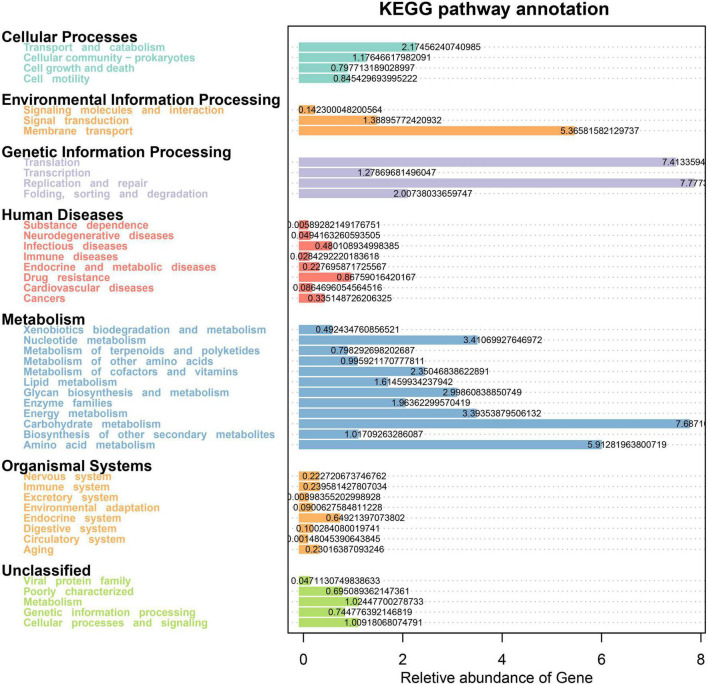
Tax4Fun predictive analysis of microbial function showing relative abundance of assigned KEGG pathway for the subsystem levels. The *X*-axis represents the relative abundance of pathways, and the *y*-axis represents the KEGG term.

We further performed functional pathway prediction based on the 16S metagenomic data and discovered several enriched metabolic pathways in the present study. The dominant KEGG pathway-related processes in all four treatment groups include Metabolism (Carbohydrate, Amino acid, Nucleotide, Energy, Glyca biosynthesis, Cofactors, and vitamins), Genetic information processing (Replication and repair, Translation, Folding, sorting, and degradation), Environmental Information Processing (Membrane transport and Signal transduction), and Cellular Processes (Transport and catabolism, Cellular community-prokaryotes). In addition to this, the genes belonging to Organismal Systems and Human Diseases pathways were also predicated (Figure 7).

**FIGURE 8:**
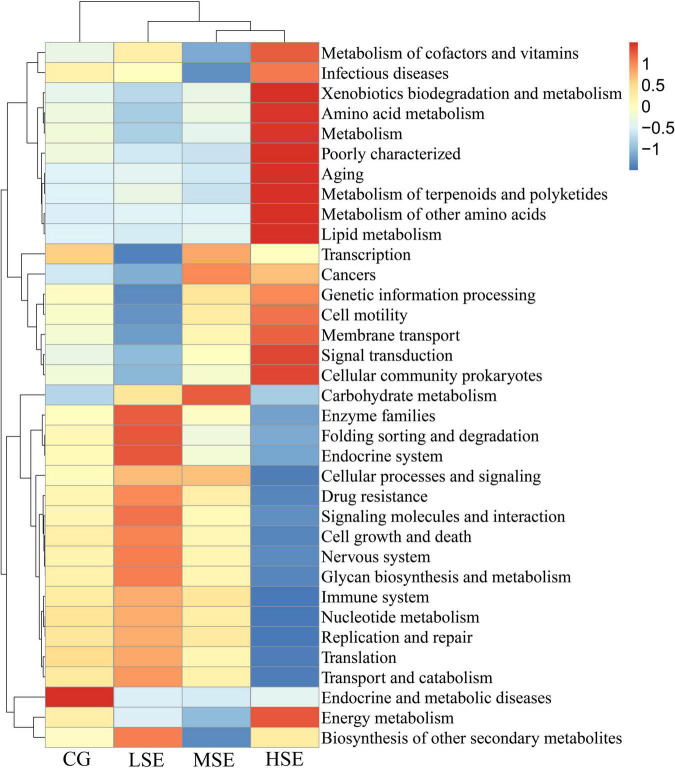
Heatmap analysis of top 35 predictive functional KEGG pathways as determined by the relative abundance of predictive functional KEGG pathway level 2. The vertical axis is sample information, the horizontal axis is species annotation information. The heatmap in the middle corresponds to the standardized Z-score of the relative abundance of each row functional pathways.

**FIGURE 9:**
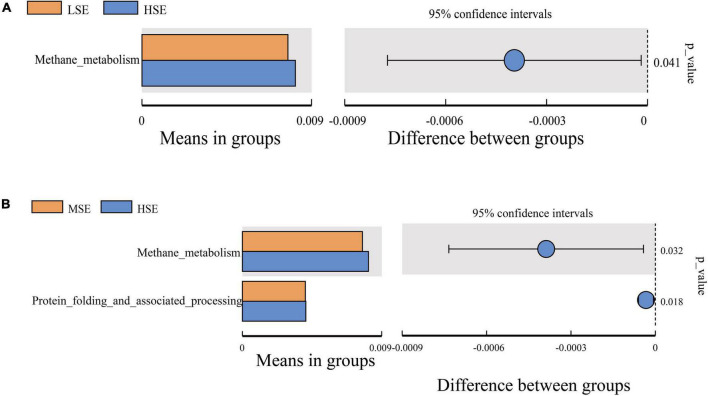
Comparison of the enrichment of KEGG pathway level 3 between LSE and HSE group **(A)**, MSE and HSE group **(B)**.

For differentially predicted pathway comparison analysis, we made a heatmap based on the top 35 pathway information at KEGG pathway level 2, and the results showed that the abundances of Xenobiotics biodegradation and metabolism, Amino acid metabolism, Metabolism, Poorly characterized, Aging, Metabolism of terpenoids and polyketides, Metabolism of other amino acids, Lipid metabolism, Signal transduction, Cellular community prokaryotes and Energy metabolism were all correlated positively with the HSE group (high level Se); Enzyme families (including peptidases and amino acid related enzymes), Folding sorting and degradation, Endocrine system, Signaling molecules and interaction, Cell growth and death, Nervous system, Glycan biosynthesis and metabolism, and Biosynthesis of other secondary metabolites were correlated positively with the LSE group (low level Se); Carbohydrate metabolism and Endocrine and metabolic diseases were correlated positively with the MSE group (medium level Se) and CG group (control), respectively (Figure 8). To investigate pathways that differ significantly between each two groups, we performed the t-test to analyze the comparison between the four experimental groups at KEGG pathway level 3. Notably, the proportion of predicted pathway of Methane metabolism in HSE was significantly higher than that of MSE and LSE groups, respectively (*P* < 0.05) (Figure 9). Additionally, the Kruskal−Wallis rank sum test showed that a sum of twenty-eight pathways were significantly different among CG, LSE, MSE, and HSE groups (*P* < 0.05, Supplementary Table 4).

## Discussion

The main objective of this study was to evaluate the effect of dietary addition of SeHLan on the growth performance, nutrient digestibility, and rumen bacterial community composition in Shaanbei white cashmere wether goats. To comprehend this, we conducted 70-day feed experiment and 11-day digestibility trial with different SeHLan supplementation (control, low, medium and high Se concentrations) and the rumen bacterial population was analyzed using the sequence of V3−V4 hypervariable region of 16S rRNA gene. There are many reports that studied the effect of dietary Se supplementation on the growth performance of ruminants, but the results are not consistent. Several research results showed that diet Se supplementation has no significant effect on growth or weight gain in cattle, goats, or sheep. Gunter et al. (2003) reported that supplement of inorganic Se (sodium selenite) and organic Se (SeY) has no effect on growth performance of calves. Similar results were also reported by Chorfi et al. (2011), who reported that dietary Se addition has no effect on the DMI and ADG in heifers. Se affects the growth rates by activating the conversion of tetraiodothyronine (T4) to triiodothyronine (T3) that depends on the enzyme 5-iodothyronine deiodinase (Chadio et al., 2006), which is a Se-dependent selenoprotein, and it is also the last selenoprotein whose activity is affected in the state of Se deficiency (Thompson et al., 1995). This partly explain why Se supplementation has no effect on animal growth performance (body weight, daily gain, and nutrient digestibility). On the other hand, the response to Se supplementation also depends on the different animal species, the form of Se supplementation (different organic or inorganic Se), and the current Se status of livestock (low or sufficient). However, the results are in contrast with the report by Wichtel et al. (1996), who investigated that using intra-ruminal bolus in 5-month calves increased ADG by 20%. Shi et al. (2011), Wang et al. (2019) reported that the sheep diet supplemented nano-Se or SeY could increase DMI and decrease FCR, improve the apparent digestibility of CP, NDF, and ADF. Xun et al. (2012), Taheri et al. (2016) also proved that dietary supplementation with Nano-Se or SeY improved DMI and nutrient digestibility significantly in early lactation of Iranian goats or sheep. Similarly, supplementation of Se to the diet of steers increased their growth rate by 0.041 to 0.060 kg/day (Gleed et al., 1983). Our results are consistent with most reports that dietary Se supplementation could improve animal’s growth performance. FCR and nutrient digestibility are methods to directly assess feed conversion efficiency. The smaller FCR value and higher apparent digestibility of nutrients, including DM, CP, DNF, and ADF, indicating that the higher efficiency of feed conversion and utilization, ultimately promote animal growth and development. In the present study, we found that dietary supplementation of SeHLan could significantly increase digestibility of DM, CP, NDF, and ADF, and the SeHLan addition groups have a lower FCR values, which suggest that SeHLan addition could improve the nutrients utilization, and increase Shaanbei white cashmere goats production. Previous studies have confirmed that dietary addition of nano-Se or SeY significantly increased the growth and activity of rumen bacteria in sheep, thereby improving rumen nutrient digestibility and promoting sheep growth (Wang et al., 2019; Cui et al., 2021). Therefore, we speculate that the addition of SeHLan to the diet changed the structure and function of the rumen bacterial population of Shaanbei white cashmere goats, thereby improving the feed utilization efficiency and promoting production.

We conducted 16s RNA high-throughput sequencing to study the effect of dietary SeHLan supplementation on the structure of rumen microbiome in Shaanbei white cashmere wether goats. The obtained bacteria diversity and richness indices of receiving SeHLan-supplemented goats were all higher than those of control group. However, the α-diversity indices (Observed species, Shannon, Simpson, Chao1, ACE, Good’s coverage, Pielou_e) were not significantly different among four treatment groups, indicating that SeHLan supplementation did not affect the diversity and abundance of rumen bacterial communities of Shaanbei white cashmere wether goats. Studies of growing puppies (Pereira et al., 2020) and Tibetan Sheep (Cui et al., 2021) reported that the alpha-diversity index of the gut and rumen bacterial community, including diversity and abundance, was also not affected by the selenite- or SeY-supplemented diets, those results are consistent with ours.

In the present study, the dominant abundance of bacterial phyla was Bacteroidetes and Firmicutes, followed by Euryarchaeota. However, contrary to our results, Cui et al. (2021) reported that ruminal most abundant phylum was Firmicutes, Bacteroidetes, and Proteobacteria, when SeY added to Tibetan sheep’s diet. The reasons for this may be related to different experimental animals (goats vs. sheep) and different forms of Se supplementation (SeHLan vs. SeY). Bacteroidetes are the Gram-negative anaerobic bacteria and regarded as specialists for the degradation of high-molecular-weight organic matter, i.e., proteins and carbohydrate and well reported for their role in starch, pectin, and xylan digestion (Parmar et al., 2014; Koringa et al., 2019), while Firmicutes for their role in energy utilization (Wu et al., 2011; Chen et al., 2015). In this study, we found that with the increase of dietary SeHLan addition, the relative abundances of Bacteroidetes and Firmicutes were all decreased, and the relative abundances of LSE and MSE groups were all higher than other two groups for those two phyla, while no significant difference was observed (*P* < 0.05), indicating that the dietary supplementation of lower (LSE) or moderate level SeHLan (MSE) may be more suitable for the survival of the phyla Bacteroidetes and Firmicutes for utilization of nutrients, such as carbohydrates, protein, and energy. Notably, the relative abundances of the phylum Euryarchaeota, family Methanobacteriaceae and genera *Methanobrevibacter* were all significantly affected by dietary SeHLan supplementation (*P* < 0.05). The *Methanobrevibacter* affiliated to the phylum Euryarchaeota, the only known microorganisms capable of methane production, is the main rumen methanogens (Hook et al., 2010). Ruminants produce methane through the rumen normal process of feed anaerobic fermentation and digestion, and large amount of methane is exhaled into the air not only causes global greenhouse gases in the atmosphere but also wastes 2–12% feed-derived energy for livestock (Johnson and Ward, 1996). Previous study on sheep confirmed that the selenate (inorganic Se) diet tended to decrease the concentrations of CH_4_ and CO_2_, while the SeY (one kind of organic Se) diet increased concentrations of CH_4_ and CO_2_ when compared with the control diet and the selenate (inorganic Se) diet (Miltko et al., 2016), indicating that organic Se and inorganic Se have different effects on the rumen methanogenesis. Furthermore, Pan et al. (2021) reported that CH_4_ output in the proportions of GE, DE, and ME intake for 6 μg/kg body weight (BW) of Se addition was significantly lower than 0, 3, 9, and 12 μg/kg BW Se supplemented groups. This is consistent with our results that the relative abundance of methanogens (mainly phylum Euryarchaeota, genera *Methanobrevibacter* belong to family Methanobacteriaceae) in the rumen of HSE and control group (CG) were all significantly higher than that in the LSE and MSE groups. We speculated that high dietary SeHLan supplementation mainly contributed to the ruminal methane production causing feed energy loss, while low and moderate SeHLan supplementation appear to be more beneficial for efficient feed energy utilization. Furthermore, dietary SeHLan supplementation significantly affected the relative abundance of Synergistetes in the rumen of different treatment groups, and the abundance of Synergistetes gradually increased with increasing SeHLan addition. Synergistetes have been found in many anaerobic environments, including the gastrointestinal tract of animals and insects, wastewater treatment systems, soil, oil wells, and so on, and have also been linked to periodontal disease (Hugenholtz et al., 2009). A previous study showed that Synergistetes are anaerobic amino acid degraders, which involved in the utilization and conversion of amino acids in natural anaerobic ecosystems (Godon et al., 2005). Our results imply that Synergistetes are primarily responsible for rumen Se utilization; however, this hypothesis requires further research. In addition, received SeHLan addition groups had lower abundance of Actinobacteria than control group. Actinobacteria are gram-positive, regular, though infrequent, members of the rumen microflora, representing up to 3% of total rumen bacteria (Sulak et al., 2012), and lack of ecological and biological information on rumen actinomycetes. Further studies to investigate the functions of taxa within Actinobacteria in the rumen of goats are required to determine whether these taxon are linked to Se metabolism in ruminants. At the family level, with the increase of dietary SeHLan supplementation level, the relative abundance of *Selenomonadaceae* and *Bifidobacteriaceae* significantly increased first and then decreased, whereas the relative abundance of *Christensenellaceae* significantly decreased first and then increased. Structural changes reported in the taxon *Selenomonadaceae* and their clinical and physiological significance were rarely studied in ruminants. A previous study showed that kefir, as a probiotic food supplement, consumption significantly increased the relative abundance of the family *Selenomonadaceae* in dogs (Kim et al., 2019). *Bifidobacteriaceae* (phylum Actinobacteria) are isolated from various ecological niches, including the gastrointestinal tract of humans and various other mammals, insect gut, oral cavity, and sewage (Kim et al., 2019). As a gut microbiota, *Bifidobacteriaceae* exhibit probiotic or health-promoting effects on the host, and also affect inflammatory state through alterations in gut microbiota of human (Cruz-Mora et al., 2014). Khiaosa-Ard et al. (2020) reported that the rumen acidotic condition shaping the microbial community and function with the highly abundant families like Bifidobacteriaceae, Lactobacillaceae, and Prevotellaceae thrived under the stress (Khiaosa-Ard et al., 2020). Previous studies showed that the family *Christensenellaceae* (phylum Firmicutes), as a health-related group, in the human is negatively related to visceral fat mass or host body mass index (BMI) and indicated as a marker of lean phenotype (Goodrich et al., 2014; Beaumont et al., 2016; Waters and Ley, 2019). In this study, we found that supplemented medium SeHLan seems to be more suitable for the growth of *Selenomonadaceae* and *Bifidobacteriaceae*, while supplemented high SeHLan suitable for the *Christensenellaceae* growth. Those results are consistent with the aforementioned experimental results that dietary supplementation of low (LSE) and medium (MSE) SeHLan could effectively increase the body weight, ADG, and improve food conversion ratio of Shaanbei white cashmere wether goats, while high SeHLan (HSE) is not beneficial for cashmere goat growth.

At the genus level, the relative abundances of *Methanobrevibacter*, *Quinella*, *unidentified_F082*, *SP3-e08*, *Christensenellaceae_R-7_group*, *Veillonellaceae_UCG-001*, and *Succinivibrionaceae_UCG-002* were all significantly affected by dietary SeHLan supplementation. *Quinella* has been reported to be the mainly involved degradation of carbohydrates in the rumen and found to possess the metabolism of utilizing lactate similar to *Selenomonas ruminantium* (Krumholz et al., 1993; Belenguer et al., 2010). Therefore, the higher proportion of Quinella may affect the rumen readily fermented carbohydrates utilization efficiency in Shaanbei white cashmere wether goats. In this feeding experiment, the relative abundances of this genera in SeHLan supplementation groups, including LSE, MSE and HSE, were all significantly higher than that in the control group (CG), and the LSE group had the highest content, indicating that dietary supplementation with SeHLan promoted rumen carbohydrate metabolism in Shaanbei white cashmere wether goats. *Christensenellaceae R7 group* (family *Christensenellaceae*) have the capable of secretin*g*α*-*arabinosidase, β-glucosidase, and β-galactosidase, which promotes degradation of hemicellulose and cellulose, to improve the feed utilization efficiency (Perea et al., 2017). However, the abundance of Christensenellaceae was inversely associated with body weight (Waters and Ley, 2019). In this study, with the increase of dietary SeHLan addition, the relative abundance of *Christensenellaceae R7* gradually increased, which was significantly lower than that of the control group. The decreased relative abundance of *Christensenellaceae R7* may increase the weight gain of livestock. We speculate that decreased relative abundance of *Christensenellaceae R7* may be associated with increased body weight for the Shaanbei white cashmere wether goats. Those results support the hypothesis that dietary SeHLan supplementation reduces the abundance of *Christensenellaceae R7*, which is beneficial for the growth of Shaanbei white cashmere wether goats. Specifically, dietary supplementation with low or medium level of SeHLan could possibly help promote growth of Shaanbei white cashmere wether goats. However, high amounts of SeHLan supplementation may be disadvantageous in promoting the growth of Shaanbei white cashmere wether goats. *Veillonellaceae_UCG-001* belongs to the phylum Firmicutes and has the function of degrading and utilizing cellulose. Chen et al. (2021) reported that supplementation with probiotics (*Bacillus licheniformis*, *Bacillus subtilis*, and *Lactobacillus plantarum* with the ratio of 1:1:0.5) and Chinese medicine polysaccharides (mixture of *L. barbarum* and *A. membranaceus* with the ratio of 2:1, contained 114.7 mg/g of polysaccharides) changed the rumen microbial fermentation mode, with the relative abundance of *Veillonellaceae_UCG-001* in the probiotics group was increased compared with the control group, and ultimately improved the growth performance of lambs. In the present study, the relative abundance of *Veillonellaceae_UCG-001* significantly decreases when fed the diet added SeHLan, indicating that diet supplementation with low and medium SeHLan might improve the cellulose digestion and feed energy utilization efficiency. In terms of relative abundance, the propionate-producing bacteria *Succinivibrionaceae UCG-002* is an crucial member of the family Succinivibrionaceae, which compete with methanogens to produce succinate and propionate instead of methane using hydrogen as a substrate, thereby reducing methane emissions and improving feed energy utilization (Pope et al., 2011; Danielsson et al., 2017). Previous researches found that the *Succinivibrionaceae_UCG-002* has a positive correlation with rumen TVFA and acetate production in dairy cattle (Hao et al., 2021) or feed efficiency in sheep (Zhang et al., 2021). In our trial, the relative abundance of *Succinivibrionaceae_UCG-002* in the SeHLan added groups was significantly higher than control group, and the LSE and MSE groups had the highest abundance of *Succinivibrionaceae_UCG-002*, while the CG and HSE groups had the lowest abundance of *Succinivibrionaceae_UCG-002*. This bacterial population changes pattern was in contrary to the aforementioned result that the CG and HSE group had the highest abundance of methanogens (mainly phylum Euryarchaeota, genera *Methanobrevibacter* belong to family Methanobacteriaceae), while LSE and MSE groups had the lowest abundance of methanogens. We could speculate that low- or medium-level SeHLan addition reduced the rumen methane production and improve feed utilization. However, high-dose SeHLan supplementation increased methane production, which is not conducive to improving animal production. However, the specific mechanism needs to be further explored.

Based on Tax4Fun-predictive analysis of microbial function revealed that several metabolic pathways were enriched in SeHLan supplemented experimental groups, including Xenobiotics biodegradation and metabolism, Amino acid metabolism, Metabolism of terpenoids and polyketides, Metabolism of other amino acids, Lipid metabolism and Energy metabolism were all mostly enriched in the HSE group. Carbohydrate metabolism was mostly enriched in the MSE group, Enzyme families, Folding sorting and degradation, Endocrine system, Signaling molecules and interaction, Cell growth and death, Nervous system, Glycan biosynthesis and metabolism were mostly enriched in the LSE group. Those results suggest that dietary SeHLan supplementation enhanced the enrichment of genes related to energy metabolism, amino acid metabolism, and carbohydrate metabolism, as well as peptidases and amino-acid-related enzymes. The enrichment of these metabolism related pathways might contribute to the growth and weight gain of growth period Shaanbei white cashmere wether goats. These findings are consistent with our aforementioned experimental results that dietary SeHLan supplementation promotes growth performance and improves apparent digestibility of nutrients in Shaanbei white cashmere wether goats. It is worth noting that the significant enrichment of “Methane metabolism” pathway, which belong to the “Energy metabolism,” in the CG and HSE group may be due to the presence of high abundance of major methanogens of phylum Euryarchaeota and genera *Methanobrevibacter* belong to family Methanobacteriaceae that produce methane. This pathway directly related to methane production when energetically favorable electron acceptors, such as oxygen, nitrate, sulfate, and iron, are absent or depleted, methanogenic archaea ferment organic matter to form methane under anaerobic conditions (Evans et al., 2019). Methane metabolism in Euryarchaeota has been proved to anaerobically produce or consume methane through the key enzymes of methyl-coenzyme M reductase (MCR) complex (Evans et al., 2019). Notably, some human-related metabolic pathways were also identified in this study, which may be due to annotation defects in the KEGG database and/or homology between bacterial and human metabolic pathways. These pathways were not discussed in this study. In addition, SeHLan addition may affect rumen health and animal growth by regulating the interaction among various microorganisms in the rumen and microbial metabolites, but the specific mechanism is still unclear. Therefore, further high-throughput sequencing experiments such as metagenomic, transcriptomics, and metabolomic approach are required to verify the functions and roles of those bacteria enriched in the rumen due to dietary SeHLan supplementation in Shaanbei white cashmere wether goats growth.

## Conclusion

This study revealed that dietary addition of SeHLan changed the rumen microbial community structure and exerted a positive effect on the rumen microbial metabolic function of Shaanbei white cashmere wether goats, which finally promoted cashmere goat growth, improved the weight gain and feed utilization efficiency. Many rumen bacterial abundances differed significantly with variations in SeHLan dose, and predictive analysis of microbial function suggested that these microbial communities may have specific functions in rumen metabolism; however, further studies are needed to determine Shaanbei white cashmere goat rumen microbial composition, structure and function using metagenomics, metabolomics, and metatranscriptomics methods. Considering the risk of Se toxicity and the results of this experimental study, we recommend an optimal dietary addition of Se level is 0.6 mg/kg DM with organic Se in the form of SeHLan for the Shaanbei white cashmere goats.

## Data availability statement

The datasets presented in this study can be found in online repositories. The names of the repository/repositories and accession number(s) can be found below: https://www.ncbi.nlm.nih.gov/, PRJNA835512.

## Ethics statement

The animal study was reviewed and approved by Animal Care and Experimental Procedures were approved by the Animal Care Committee of Yulin University (Yulin, China) and were under the university’s guidelines for animal research (file no.: YLU2021-2).

## Author contributions

L-PL designed the study, performed the data analysis, wrote the manuscript, and funded the acquisition. LQ helped to carry out feeding experiments. TL helped to collect the rumen samples and extract the DNA. All authors contributed to the article and approved the submitted version.
