# Supplementary material for: Supplemental dietary Selenohomolanthionine affects growth and rumen bacterial population of Shaanbei white cashmere wether goats
Source: Front Microbiol. 2022 Oct 20;13:942848. doi: 10.3389/fmicb.2022.942848 (PMC9632625; doi:10.3389/fmicb.2022.942848)
Supplement: Supplementary file 1 [file Data_Sheet_1.doc]

**Supplementary Tables**

**Table S1. The relative abundance (%) of rumen microbiota at phyla level among four SeHLan supplemented groups (average relative abundance > 0.1% for at least one group).**

| Phylum | Treatments^1^ | | | | SEM | *P*-value |
| --- | --- | --- | --- | --- | --- | --- |
|  | CG | LSE | MSE | HSE |  |  |
| Bacteroidetes | 39.47 | 47.75 | 43.43 | 34.94 | 2.79 | 0.383 |
| Firmicutes | 29.75 | 35.72 | 32.09 | 30.29 | 2.14 | 0.758 |
| Euryarchaeota | 15.58 a | 3.69 b | 5.62 b | 17.13 a | 1.49 | 0.001 |
| unidentified_Bacteria | 4.59 ab | 3.98 b | 6.62 ab | 9.52 a | 0.94 | 0.378 |
| Proteobacteria | 1.24 | 2.44 | 4.13 | 1.99 | 0.53 | 0.501 |
| Actinobacteria | 3.07 a | 0.29 b | 0.52 | 0.44 b | 0.34 | 0.004 |
| Synergistetes | 0.18 a | 0.73 | 1.56 b | 1.62 b | 0.26 | 0.011 |
| Others | 6.12 a | 5.39 ab | 6.02 ab | 4.05 b | 0.36 | 0.195 |

^1^CG, LSE, MSE, and HSE, treatment groups supplemented with SeHLan at 0, 0.3, 0.6, and 1.2 mg/kg DM,

respectively.

^a,b^Within rows, means without a common superscript differ (*P* < 0.05).

**Table S2.** **The relative abundance (%) of rumen microbiota at family level among four SeHLan supplemented groups (average relative abundance >1% for at least one group).**

| Family | Treatments^1^ | | |  | SEM | *P*-value |
| --- | --- | --- | --- | --- | --- | --- |
|  | CG | LSE | MSE | HSE |  |  |
| Prevotellaceae | 24.02 | 39.08 | 32.17 | 25.48 | 2.54 | 0.240 |
| Selenomonadaceae | 3.68 | 21.21 | 13.13 | 6.73 | 2.16 | 0.002 |
| Methanobacteriaceae | 15.58 a | 3.69 b | 5.62 b | 17.13 a | 1.49 | 0.001 |
| Bifidobacteriaceae | 1.35 a | 1.13 | 4.59 b | 7.75 c | 0.93 | 0.012 |
| F082 | 6.96 | 2.05 | 1.73 | 2.77 | 1.36 | 0.639 |
| Acidaminococcaceae | 4.65 | 1.83 | 2.26 | 1.28 | 0.69 | 0.167 |
| Rikenellaceae | 4.37 | 3.86 | 2.79 | 2.54 | 0.64 | 0.551 |
| Lachnospiraceae | 5.78 | 3.47 | 4.69 | 6.71 | 0.66 | 0.218 |
| Succinivibrionaceae | 0.69 b | 2.18 | 3.88 a | 1.32 | 0.54 | 0.060 |
| Ruminococcaceae | 3.83 | 2.32 | 2.93 | 4.95 | 0.56 | 0.738 |
| Bacteroidales_RF16_group | 1.65 | 0.94 b | 4.02 a | 1.59 | 0.48 | 0.212 |
| Christensenellaceae | 5.37 a | 1.93 b | 3.27 | 2.27 b | 0.44 | 0.014 |
| Others | 24.32 | 16.31 | 18.92 | 17.22 | 1.09 | 0.073 |

^1^CG, LSE, MSE, and HSE, treatment groups supplemented with SeHLan at 0, 0.3, 0.6, and 1.2 mg/kg DM, respectively

^a,b^Within rows, means without a common superscript differ (*P* < 0.05).

**Table S3. The relative abundance (%) of rumen microbiota at genera level among four SeHLan supplemented groups (average relative abundance >1% for at least one group).**

| Phylum | Genus | Treatments^1^ | | | | SEM | *P*-value |
| --- | --- | --- | --- | --- | --- | --- | --- |
|  |  | CG | LSE | MSE | HSE |  |  |
| Bacteroidota | *Prevotella* | 20.54 b | 34.59 a | 29.48 | 22.54 | 2.46 | 0.288 |
| Euryarchaeota | *Methanobrevibacter* | 15.55 a | 3.68 b | 5.61 b | 17.10 a | 1.48 | 0.001 |
| Firmicutes | *Quinella* | 0.88 a | 14.94 b | 7.79 c | 3.72 | 2.03 | 0.001 |
| Bacteroidota | *unidentified_F082* | 5.45 a | 0.31 | 0.17 | 0.04 b | 1.29 | 0.013 |
| Firmicutes | *Succiniclasticum* | 1.28 | 1.83 | 2.26 | 4.66 | 0.69 | 0.167 |
| Bacteroidota | *SP3-e08* | 2.09 a | 0.01 b | 0.01 b | 0.03 b | 1.98 | 0.002 |
| Firmicutes | *Christensenellaceae_R-7_group* | 5.37 a | 1.93 b | 3.27 | 2.27 | 0.44 | 0.014 |
| Bacteroidota | *Rikenellaceae_RC9_gut_group* | 2.25 | 3.83 | 2.77 | 2.48 | 0.36 | 0.422 |
| Firmicutes | *Ruminococcus* | 2.55 | 1.22 | 1.57 | 3.59 | 1.29 | 0.690 |
| Proteobacteria | *Succinivibrio* | 0.55 | 1.49 | 2.10 | 0.86 | 0.37 | 0.176 |
| Actinobacteria | *Bifidobacterium* | 0.39 | 0.22 | 0.45 | 1.75 | 0.29 | 0.100 |
| Firmicutes | *Veillonellaceae_UCG-001* | 0.84 a | 2.82 b | 1.43 | 0.60 a | 0.28 | 0.018 |
| Proteobacteria | *Succinivibrionaceae_UCG-002* | 0.04 a | 0.39 b | 1.68 c | 0.16 | 0.26 | 0.010 |
| Firmicutes | *Lachnospiraceae_NK3A20_group* | 1.66 | 0.45 | 0.59 | 1.19 | 0.24 | 0.462 |
|  | *Others* | 38.15 | 32.26 | 40.82 | 41.39 | 1.59 | 0.189 |

^1^CG, LSE, MSE, and HSE, treatment groups supplemented with SeHLan at 0, 0.3, 0.6, and 1.2 mg/kg DM, respectively.

^a,b^Within rows, means without a common superscript differ (*P* < 0.05).

**Table S4.** **Functional predictions for rumen microbiota among four SeHLan supplemented groups with significantly different KEGG pathways level 2.**

| KEGG Pathway | | Treatments^1^ | | | | SEM | *P*-value |
| --- | --- | --- | --- | --- | --- | --- | --- |
|  |  | CG | LSE | MSE | HSE |  |  |
| Organismal Systems | Aging | 0.76 | 0.71 | 0.70 | 0.72 | 0.06 | 0.030 |
|  | Circulatory system | 0.012 | 0.001 | 0.002 | 0.002 | 0.001 | 0.000 |
|  | Digestive system | 0.25 a | 0.37 b | 0.32 | 0.32 | 0.013 | 0.006 |
|  | Nervous system | 0.665 a | 0.722 b | 0.698 | 0.699 | 0.006 | 0.002 |
|  | Excretory system | 0.041 a | 0.022 b | 0.025 | 0.023 b | 0.002 | 0.002 |
| Metabolism | Amino acid metabolism | 18.15 | 18.32 | 18.33 | 19.10 | 0.09 | 0.001 |
|  | Glycan biosynthesis and metabolism | 8.41 a | 10.13 b | 9.45 | 9.49 | 0.19 | 0.006 |
|  | Carbohydrate metabolism | 23.05 | 24.15 | 23.88 | 23.99 | 0.04 | 0.05 |
|  | Metabolism of other amino acids | 3.05 | 3.07 | 3.06 | 3.27 | 0.03 | 0.000 |
|  | Lipid metabolism | 4.76 b | 4.79 b | 4.78 b | 5.84 a | 0.12 | 0.000 |
|  | Nucleotide metabolism | 9.92 a | 11.08 b | 10.78 | 10.85 | 0.12 | 0.006 |
|  | Enzyme families | 5.927 | 6.357 | 6.129 | 6.133 | 0.06 | 0.050 |
|  | Xenobiotics biodegradation and metabolism | 1.18 | 1.37 | 1.35 | 2.24 | 0.103 | 0.001 |
|  | Metabolism of terpenoids and polyketides | 2.37 b | 2.39 b | 2.44 | 2.77 a | 0.04 | 0.001 |
| Human Diseases | Cardiovascular diseases | 0.264 a | 0.273 b | 0.272 | 0.271 | 0.034 | 0.017 |
|  | Neurodegenerative diseases | 0.202 | 0.135 | 0.141 | 0.139 | 0.006 | 0.000 |
|  | Immune system | 0.67 a | 0.79 b | 0.77 b | 0.76 | 0.012 | 0.001 |
|  | Drug resistance | 2.45 a | 2.91 b | 2.75 | 2.74 | 0.048 | 0.004 |
| Unclassified | Poorly characterized | 2.13 | 2.11 | 2.15 | 2.29 | 0.01 | 0.000 |
|  | Cellular processes and signaling | 3.03 a | 3.22 b | 3.20 b | 3.16 | 0.019 | 0.002 |
|  | Metabolism | 3.06 b | 3.13 b | 3.16 | 3.46 a | 0.038 | 0.001 |
| Cellular Processes | Cell growth and death | 2.32 a | 2.62 b | 2.50 | 2.52 | 0.17 | 0.024 |
|  | Cellular community-prokaryotes | 3.80 a | 3.58 b | 3.66 | 3.65 | 0.025 | 0.028 |
|  | Transport and catabolism | 6.38 a | 7.08 b | 6.85 | 6.88 | 0.076 | 0.006 |
| Environmental Information Processing | Signaling molecules and interaction | 0.38 a | 0.49 b | 0.44 | 0.45 | 0.01 | 0.014 |
|  | Signal transduction | 0.50 a | 0.38 b | 0.43 | 0.41 | 0.14 | 0.033 |
| Genetic Information Processing | Replication and repair | 2.26 a | 2.53 b | 2.46 | 2.47 | 0.28 | 0.005 |
|  | Translation | 0.221 a | 0.238 b | 0.234 | 0.235 | 0.206 | 0.040 |

^1^CG, LSE, MSE, and HSE, treatment groups supplemented with SeHLan at 0, 0.3, 0.6, and 1.2 mg/kg DM, respectively.

^a,b^Within rows, means without a common superscript differ (*P* < 0.05).
